# Brassinosteroids Render Cell Walls Softer but Less Extensible in Growing Arabidopsis Hypocotyls

**DOI:** 10.3390/plants14020176

**Published:** 2025-01-10

**Authors:** Dmitry V. Suslov, Alexandra N. Ivanova, Daria Balcerowicz, Mariia S. Tarasova, Nuria K. Koteyeva, Kris Vissenberg

**Affiliations:** 1 Department of Plant Physiology and Biochemistry, St. Petersburg State University, 199034 St. Petersburg, Russia; 2 Laboratory of Anatomy and Morphology, Komarov Botanical Institute of Russian Academy of Sciences, 197376 St. Petersburg, Russia; 3 Research Park, St. Petersburg State University, 199034 St. Petersburg, Russia; 4 Integrated Molecular Plant Physiology Research, Biology Department, University of Antwerp, 2020 Antwerpen, Belgium

**Keywords:** brassinosteroids, cell wall, growth, biomechanics, creep, extensibility, cellulose, microfibrils, expansins

## Abstract

Cell wall extensibility is a key biophysical characteristic that defines the rate of plant cell growth. It depends on the wall structure and is controlled by numerous proteins that cut and/or (re)form links between the wall constituents. Cell wall extensibility is currently estimated by different in vitro biomechanical tests. We used the creep method, in which isolated cell walls are extended under a constant load and their time-dependent deformation (creep) is recorded to reveal the biophysical basis of growth inhibition of *Arabidopsis thaliana* hypocotyls in the presence of 24-epibrassinolide (EBL), one of the most active natural brassinosteroids. We found that EBL rendered the walls of hypocotyl cells softer, i.e., more deformable under mechanical force, which was revealed using heat-inactivated cell walls to eliminate endogenous activities of cell-wall-loosening/tightening proteins. This effect was caused by the altered arrangement of cellulose microfibrils. At the same time, EBL made the walls less extensible, which was detected with native walls under conditions optimized for activities of endogenous cell-wall-loosening proteins. These apparently conflicting changes in the wall mechanics can be an adaptation by which EBL enables plant cells to grow under stress conditions.

## 1. Introduction

Brassinosteroids (BS) are a class of phytohormones that regulate plant growth, morphogenesis and stress responses [1]. Many aspects of BS action in plants are mediated by rigid but extensible cell walls that surround each plant cell [2]. The walls define morphogenesis and cell expansion rate via their extensibility, i.e., the ability to increase in surface area irreversibly during growth [3,4]. Plant cell wall extensibility depends on the chemical structure of constituent polymers, their orientation and numerous enzymic and non-enzymic cell-wall-loosening and -tightening proteins that continuously break and/or form covalent and non-covalent bonds between its components [5,6].

We have studied BS effects on plant shoot posture and gravitropism and found that 24-epibrassinolide (EBL), one of the most active natural brassinosteroids, and brassinazole (BRZ), a specific inhibitor of BS biosynthesis, exerted opposite effects on the percentage of upright hypocotyls in etiolated Col-0 *Arabidopsis thaliana* seedlings grown on horizontal Petri plates [7]. Exogenous EBL decreased the percentage of upright hypocotyls in a wide range of concentrations [7,8]. This effect resulted from impaired mechanics of cell walls interfering with their ability to keep hypocotyls upright in the gravity field [7]. We revealed the above-mentioned mechanical change in vitro as an increase in the rate of cell wall creep (i.e., its time-dependent irreversible deformation under a constant load) in a neutral pH buffer [7,9]. Interestingly, the increased creep rate in vitro was observed for hypocotyls demonstrating growth inhibition in vivo in the presence of EBL [9]. This was very surprising because creep rate is considered as a good estimate for cell wall extensibility [3,10], and as such it usually correlates positively with in vivo growth rate in plant organs [11]. Explaining the discrepancy between creep rate and growth rate in the presence of EBL would improve our understanding of biophysical mechanisms that control plant cell wall expansion.

We have also investigated biochemical and structural changes in the walls accompanying the effect of EBL on *A. thaliana* hypocotyl posture [9]. Very minor alterations in the wall biochemistry were found. At the same time, EBL disorganized cellulose macrofibril orientation in the outer epidermal cell wall of hypocotyls [9], which was revealed using confocal microscopy with a cellulose-specific Pontamine Fast Scarlet 4B fluorescent dye [12]. However, the resolution of confocal microscopy is limited, such that only the largest cellulose macrofibrils could be discerned [13]. Additionally, macrofibrils do not always demonstrate the same orientation as the majority of cellulose microfibrils [14,15]. Thus, higher-resolution imaging techniques based on electron microscopy could shed more light on the involvement of cellulose arrangement in the control of growth and posture of *A. thaliana* hypocotyls in the presence of EBL.

In the present study we comprehensively characterized the wall biomechanics of hypocotyls from EBL-grown *A. thaliana* seedlings in vitro under different loads and pH values that could mimic cell wall behavior under normal physiological and stress conditions in vivo. Our aim was to reveal the biophysical mechanisms of EBL-induced growth inhibition at the cell wall level. We also tried to understand if the wall creep rate in vitro is a reliable indicator for in vivo growth rate and cell wall extensibility. Finally, we studied cellulose arrangement in the walls of hypocotyls via electron microscopy to find out whether microfibrils contribute to the EBL-induced growth inhibition.

Very interesting modifications of cell wall biomechanics in the presence of EBL were observed. *A. thaliana* hypocotyl walls became softer (i.e., more pliant or deformable to mechanical force [16]) at pH 6 or after heat inactivation. At the same time, they were less extensible (a characteristic describing the ability of the cell wall to grow) at pH 5, i.e., under conditions in which expansins, a class of cell-wall-loosening proteins, were active [17]. These effects were related to cellulose microfibril organization and could reflect the brassinosteroid-induced cell wall adaptation to stress conditions.

## 2. Results

**Figure 1:**
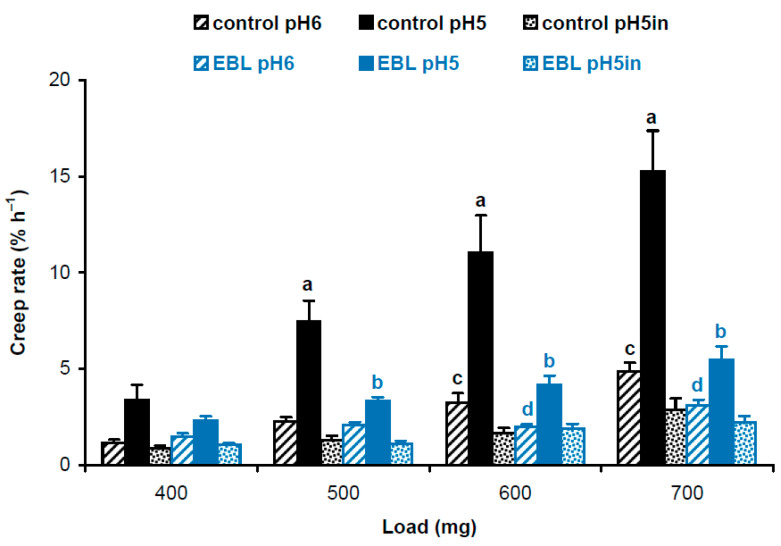
Creep rates of hypocotyl cell walls in 4-day-old etiolated Col-0 *Arabidopsis thaliana* seedlings grown with 100 nM epibrassinolide (EBL) or without it (control). Data are means ± SE (*n* = 10). Different letters ‘a’ and ‘b’ mark significant differences between control and EBL variants at pH 5 under respective loads (*p* < 0.01; Student’s *t*-test), while different letters ‘c’ and ‘d’ denote significant differences between these variants at pH 6 (*p* < 0.05; Student’s *t*-test). ‘pH5in’ refers to heat-inactivated cell walls extended at pH 5.

Four-day-old, etiolated Col-0 *A. thaliana* seedlings grown on vertical Petri plates in ES medium [18] with 100 nM EBL had significantly shorter hypocotyls compared with controls grown without EBL (11.9 ± 1.7 mm vs. 16.2 ± 3.0 mm, mean ± SD; *n* = 110; *p* < 0.0001; Student’s *t*-test). To find out the biophysical basis for this growth inhibition, we studied the biomechanics of cell walls in frozen/thawed hypocotyls using the creep method. Five-millimeter-long subapical segments of hypocotyls, including their growing zones, were extended under a range of constant loads to mimic the action of different turgor pressures on the wall. Creep curves were recorded at pH 5 and pH 6 for native cell walls retaining endogenous activities of cell-wall-loosening proteins, and at pH 5 for heat-inactivated cell walls with eliminated activities of these proteins. A strong acid-induced cell wall creep was found for EBL and control variants under all loads, when native cell walls extended at pH 5 were compared with those extended at pH 6 or heat-inactivated cell walls (Figure 1). This highlights the presence of high expansin activity in growing etiolated *A. thaliana* hypocotyls. EBL significantly decreased creep rates of native cell walls at pH 5 under 500–700 mg loads and at pH 6 under 600 and 700 mg loads. No significant differences for heat-inactivated cell walls between EBL and control variants were found (Figure 1). Thus, the reduced growth of hypocotyls in the presence of EBL was associated with decreased activities of cell-wall-loosening proteins active at pH 5, presumably expansins, and those active at pH 6.

Cell wall expansion in vivo during growth or in vitro during creep tests is proportional to the stress (= force divided by the cross-section area across which it acts) induced by turgor or a constant load, respectively. If we take, e.g., cylindrical rods made of the same material that differ in their cross-section area and extend them axially under the same load, the thinner rod will increase in length more compared with the thicker one because of the higher stress induced in the former. Hence, the data on cell wall cross-section area should be considered when interpreting the results of biomechanical tests. This would help understand if different creep rates of cell wall samples are caused by qualitative differences in their polymers, different amounts of these polymers or some combination of these two factors. So, we estimated cell wall cross-section areas in hypocotyls with a classic method based on measuring their dry weight per unit length. *A. thaliana* seedlings grown in the presence of EBL deposited thicker cell walls in hypocotyls, such that their cross-section area was 1.5-fold greater than in the control plants (Table 1). Thus, each constant load used in the creep test generated proportionally lower wall stress in EBL vs. control variants (Table 1).

**Figure 2:**
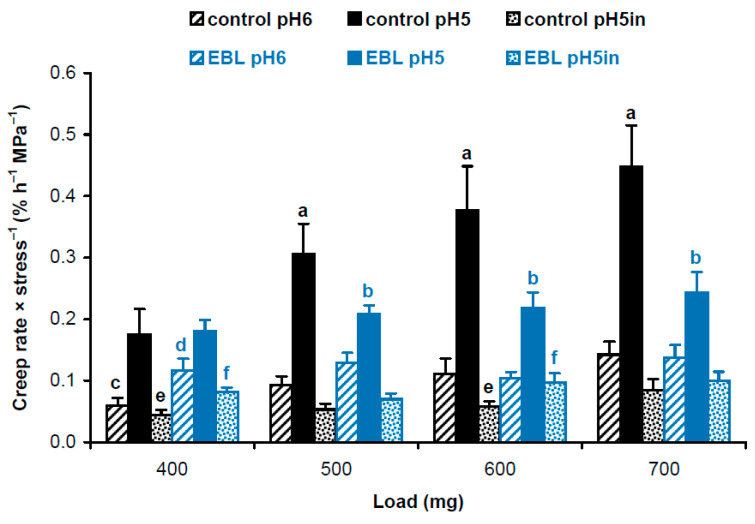
Creep rate × stress^−1^ values for cell wall material from hypocotyls of 4-day-old etiolated Col-0 *Arabidopsis thaliana* seedlings grown with 100 nM epibrassinolide (EBL) or without it (control). Creep rates (*n* = 10) and wall stress values (*n* = 4) were taken from Figure 1 and Table 1, respectively, and their ratios were calculated using a bootstrap procedure. Data are means ± SD. Different letters ‘a’ and ‘b’ mark significant differences between control and EBL variants for native cell walls at pH 5 under respective loads (*p* < 0.05; Student’s *t*-test), different letters ‘c’ and ‘d’ denote significant differences between these variants for native cell walls at pH 6 (*p* < 0.05; Student’s *t*-test) and different letters ‘e’ and ‘f’ mark significant differences between control and EBL variants for heat-inactivated cell walls at pH 5 under respective loads (*p* < 0.05; Student’s *t*-test). ‘pH5in’ refers to heat-inactivated cell walls extended at pH 5.

Considering different cross-section areas (Table 1), we compared physical properties of cell wall material in hypocotyls of EBL-grown and control plants using approaches described in our previous work [19]. We first calculated and statistically compared creep rate × stress^−1^ values using a script published in [19]. As creep rate (Figure 1) and wall stress values (Table 1) are independent variables, the script uses a bootstrap procedure where new sets of values for creep rate and wall stress are randomly resampled from the available data and the ratio creep rate × stress^−1^ is determined in each of these samples. This is done 10,000 times and the script reports the average and standard deviations of the resulting ratios. Additionally, it determines significant differences as results of *t*-tests if only two data sets are compared or *t*-tests with an FDR (false discovery rate) of 5% if more than two data sets are compared. As a result, we found that EBL rendered the wall polymeric material softer (i.e., more deformable to mechanical force [16]). This is seen as higher creep rate × stress^−1^ values for heat-inactivated cell walls under 400 and 600 mg loads and for native cell walls at pH 6 under a 400 mg load in EBL vs. control variants (Figure 2). As for native cell walls extended at pH 5, creep rate × stress^−1^ values demonstrated the same trend as creep rates being significantly lower under 500–700 mg loads when EBL variants were compared with untreated controls (Figure 2). Thus, the material of native walls was less extensible in the presence of EBL at pH 5, which implies the possible involvement of expansins.

**Figure 3:**
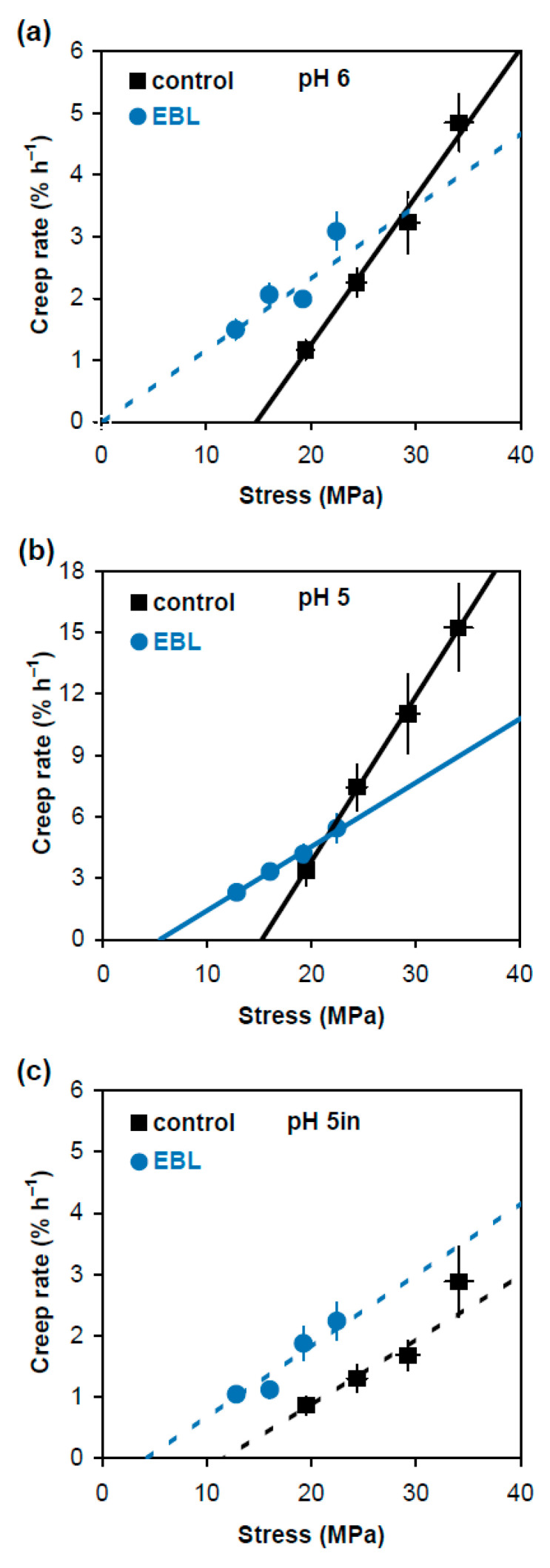
Dependence of creep rate of frozen/thawed hypocotyls of 4-day-old etiolated Col-0 *Arabidopsis thaliana* seedlings grown with 100 nM epibrassinolide (EBL) or without it (control) on the applied stress. Hypocotyls were extended at pH 6.0 (**a**); pH 5.0 (**b**); and pH 5.0 after heat-inactivation (**c**). Creep rate (*n* = 10) and stress values (*n* = 4) were taken from Figure 1 and Table 1, respectively, and are reported with their SE. The straight lines were fitted to the data using the Deming regression, taking into account the variance in both creep rate and wall stress. Solid approximating lines indicate that the Deming regression is significant while dashed approximating lines show the variants where it is nonsignificant. The slopes of the fitted straight lines correspond to in vitro cell wall extensibility (ϕ) values, and their intercepts with the *x* axis correspond to in vitro cell wall yield threshold (*y*) values. Note that the scale of the *y* axis in (**b**) differs from that in (**a**,**c**).

In an alternative approach comparing physical properties of cell wall material in EBL and control variants, we plotted creep rate values (Figure 1) against stress generated in cell walls by respective loads (Table 1). The dependencies between them were linear or close to linear (Figure 3). The approximating lines shown in Figure 3 were obtained on the basis of Deming regression. Unlike ordinary least-squares regression, it considers variation in both creep rate and wall stress to fit an optimal straight line describing their relationship. Deming regression and related statistical analyses were performed using a script published in [19]. This approach is less universal in characterizing the wall material properties than creep rate × stress^−1^ because it is valid only when Deming regression is significant (Figure 3). However, it describes the relationship between creep rate and wall stress very visually and provides two essential characteristics of cell wall mechanics: in vitro cell wall extensibility (ϕ) and in vitro cell wall yield threshold (*y*). The values of ϕ correspond to the slopes of the respective regression lines and characterize the sensitivity of creep rate to changes in wall stress. The values of *y* correspond to intercepts of the regression lines with the *x* axis and show the minimal wall stress at which creep starts. Statistical analysis of these characteristics (Table 2) demonstrated that the expansin-dependent acid-induced creep of hypocotyl cell walls was mediated by increased ϕ. At the same time, the EBL-induced growth inhibition was associated with lower *y* and ϕ values (Figure 3b, Table 2). Thus, EBL modifies the wall material such that its creep starts at wall stress values under which control cell walls do not yet extend. This could be physiologically relevant for growth under conditions of water deficit.

**Figure 4:**
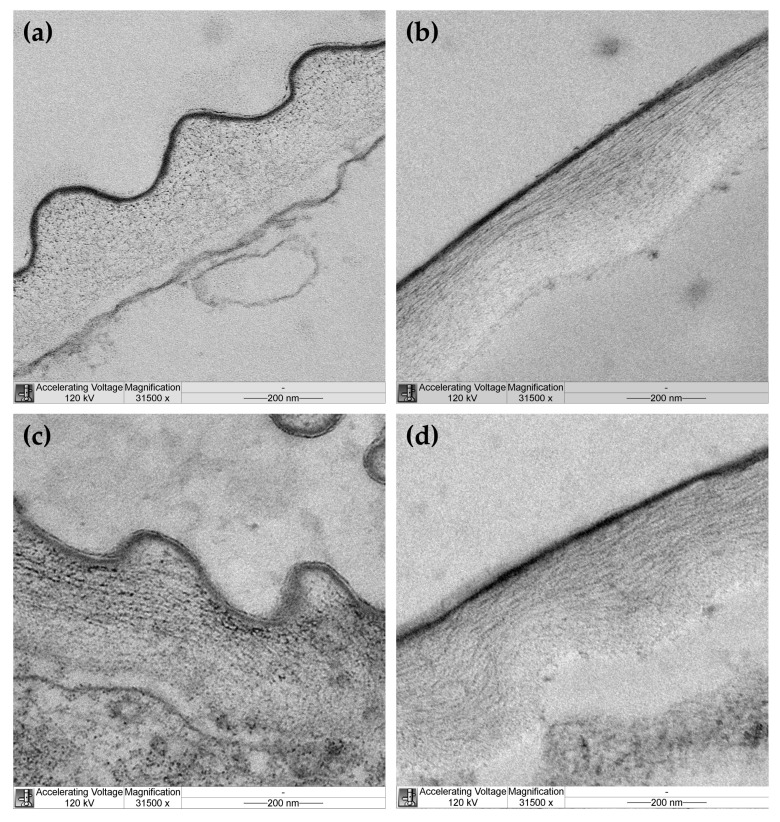
Transmission electron microscopy images of cellulose organization in the outer epidermal cell wall from hypocotyls of control (**a**,**b**) and EBL-grown (**c**,**d**) 4-day-old *Arabidopsis thaliana* plants. Transverse (**a**,**c**) and longitudinal (**b**,**d**) sections of hypocotyls taken at 3 mm under the hook. A cuticle overlying the outer face of the cell wall is seen as a thin dark layer.

Cellulose microfibril organization was studied in outer epidermal cell walls of hypocotyls from EBL-grown and control plants by transmission electron microscopy (TEM). Cellulose microfibrils were not equally well seen through the whole thickness of the cell wall in TEM images of control plants. They were clearly discernable in the outer half through the wall thickness and looked like dots on transverse sections (Figure 4a) and long well-aligned threads on longitudinal sections (Figure 4b) of hypocotyls. These findings are consistent with longitudinal alignment of microfibrils to the axis of hypocotyls in the outer part of the cell wall of control plants. Cellulose was poorly seen in the inner half of the cell wall from this variant, possibly because it was masked by amorphous pectic polysaccharides. In EBL-grown plants, cellulose microfibrils were discernable through the whole thickness of the outer epidermal cell wall (Figure 4c,d). In its outer half, microfibrils looked like dots, short or long strips on transverse sections (Figure 4c) and strips of medium or short length orientated parallel or oblique to the hypocotyl axis on longitudinal sections (Figure 4d). In the inner half of the cell wall, they were seen as rare dots or short strips of different orientations on transverse sections (Figure 4c) and very short strips of random orientations on longitudinal sections (Figure 4d). Overall, these data indicate that cellulose microfibrils are less evenly aligned in EBL-grown versus control plants with a lower proportion of axially orientated microfibrils in the outer part of the cell wall of the former compared with the latter.

## 3. Discussion

BSs are considered as growth-stimulating hormones because mutants in their biosynthesis and signaling demonstrate significant dwarfism [20]. Nevertheless, exogenous addition of BSs in a wide range of concentrations [21] or of BRZ, a BS synthesis inhibitor [9], reduced elongation of hypocotyls in etiolated Col-0 *A. thaliana* seedlings, suggesting that endogenous BS concentrations are optimal for growth in this organ. The growth inhibition by exogenous 100 nM EBL is consistent with these observations.

The role of cell wall mechanics in plant growth regulation by BSs has been addressed in prior studies that gave conflicting results. A BR-induced stimulation of soybean epicotyl elongation was accompanied by increased cell wall viscoelasticity measured by the Instron technique [22]. On the contrary, exogenous BS application to the apex of *Brassica chinensis* hypocotyls increased their growth rate without significant changes in the Instron-measured wall viscoelasticity. However, this growth response correlated with increased cell wall extensibility measured in pressure-block experiments [23]. A very recent report based on mutant analyses and modeling in *Utricularia gibba* and *A. thaliana* suggests that growth coordination between cell layers is mediated by BS-induced changes in the epidermal wall mechanics [24]. To our knowledge, the biomechanical mechanisms of plant growth inhibition in the presence of exogenous BSs have never been addressed.

We found that the growth reduction in the presence of EBL was associated with the wall thickening (Table 1) and the increased creep rate × stress^−1^ of heat-inactivated cell walls (Figure 2). The first phenomenon may reflect the fact that cell wall synthesis was not reduced by EBL, when cell elongation was inhibited. As a result, more cell wall material was accumulated per unit length of hypocotyls in EBL-grown plants. This explanation is in line with findings that cell wall synthesis and cell elongation were uncoupled during development of etiolated *A. thaliana* hypocotyls [25] or their diurnal growth oscillations in short photoperiods [11]. The second phenomenon indicates that the viscoelastic properties of cell walls have been modified in such a way that their polymeric material has become softer. Our previous studies reported very minor changes in the wall composition of *A. thaliana* hypocotyls in the presence of EBL [9]. We found only a slight increase in the content of rhamnose suggesting that the level of rhamnogalacturonan I, one of the pectic polysaccharide species, was affected. At the same time, EBL significantly randomized cellulose macrofibril orientations in the outer epidermal cell wall of hypocotyls [9]. These findings were supported at the level of cellulose microfibrils revealed using TEM in the present study (Figure 4). Cellulose orientations were less regular in the presence of EBL (Figure 4c,d) compared with the untreated control (Figure 4a,b). Importantly, the fraction of axially aligned microfibrils was lower in the EBL variant vs. the control, which makes cellulose the main candidate for the role in the observed cell wall softening (Figure 2). This is because cellulose is the strongest cell wall component [26], and its developmental reorientations parallel to the axis of maximal cell expansion were found to gradually harden the cell wall in this direction, which ultimately resulted in growth deceleration [27]. It seems that the fewer axially aligned microfibrils there are, the softer the cell wall becomes along this axis. The fact that EBL modified cellulose alignment was not surprising because it was shown that BSs reorganized cortical microtubules [28,29] that regulate the orientation of cellulose microfibrils in plant cell walls [30].

The EBL-induced growth inhibition of hypocotyls was accompanied by considerable reductions in creep rate (Figure 1), creep rate × stress^−1^ (Figure 2) and in vitro cell wall extensibility (Figure 3, Table 2) at pH 5 without heat-inactivation. This indicates the involvement of expansin proteins, the mediators of the acid growth response [17]. The acid growth theory describes the early phase of auxin-induced cell expansion postulating that auxin activates the plasma membrane H^+^-ATPases that acidify the apoplastic space, thereby increasing wall extensibility [31,32]. This effect is mediated by expansins that are activated at a pH below 5.5 and break hydrogen bonds between the wall constituents [17]. Unlike expansins, different classes of cell-wall-loosening proteins do not contribute significantly to the acid-induced creep of *A. thaliana* hypocotyls because of their low expression (yieldins), different pH-optima (the majority of xyloglucan endotransglucosylase/hydrolases, XTHs) or inability to compete with endogenous expansins (rare XTHs with a pH-optimum close to 5.0) [33]. The mechanisms related to the acid growth play an important role in the control of in vivo cell expansion in *A. thaliana* hypocotyls as their creep rates at pH 5 demonstrated very good correlations with in vivo growth rate during this organ maturation [19] or its rapid growth oscillations in diurnal cycles [11]. Because of these tight links between the mechanics of native cell walls at pH 5 and in vivo growth rates, we refer to the biomechanical effect of EBL at pH 5 without heat-inactivation as rendering the walls less extensible. The term ‘extensible’ emphasizes the likely high correlation with in vivo cell wall extensibility and in vivo growth rate. The effect of EBL on *A. thaliana* hypocotyl elongation through the mechanisms related to the acid growth response is supported by the literature data. Firstly, BSs were shown to directly regulate the activity of H^+^-ATPase via phosphorylation of one of its threonine residues [34]. Secondly, transcriptomics of dark-grown *A. thaliana* hypocotyls demonstrated that EBL induced a 3-fold decrease in the level of expansin A5 (EXPA5) gene expression [21]. Finally, randomization of cellulose microfibril arrangement in the walls of *A. thaliana* hypocotyls by specific inhibitors or mutations greatly interfered with their acid growth response [35]. This effect could involve the inaccessibility of expansins to their sites of action in the wall with the altered cellulose arrangement. As EBL also disorganized microfibril orientations (Figure 4), a similar mechanism could explain the EBL-induced growth inhibition. We thus hypothesize that EBL renders the wall less extensible by decreasing the expression of expansins and/or blocking their access to the sites of action through the EBL-induced changes in the wall architecture.

What is the physiological significance of rendering the cell wall material softer but less extensible (Figure 2 and Figure 3)? BSs were shown to increase plant tolerance to different stress conditions including drought, salinity, cold, heat and many others [1,36,37,38]. Rapid growth can be harmful for plants under severe stress. However, they should retain the ability to grow under highly unfavorable conditions to survive. The EBL-induced changes in the wall mechanics could help plants reach this compromise and grow under stress conditions. According to our data, the control cell walls did not have the ability for the long-term extension at a stress below 15.2 MPa, while their in vitro extension could start at a stress as low as 5.4 MPa in the presence of EBL (Figure 3b, Table 2). When extrapolated to in vivo conditions, this suggests that EBL could provide growth at lower turgor values, which is highly beneficial under physiological stress conditions. The energetic reserves of plants and, hence, H^+^-ATPase activities and cell wall acidification could be reduced under stress, decreasing the contribution of expansins to in vivo cell-wall-loosening. This plausible impairment of expansin activity under stress could be partially compensated by different cell-wall-loosening proteins active at more neutral pH values, such as XTHs and pectin methylesterases (PMEs) [33,39,40]. The EBL-induced changes in the wall mechanics at pH 6 demonstrate that the contribution of these different cell-wall-loosening proteins to the wall extension could also increase under low turgor values (Figure 3a). More studies are needed to reveal adaptive modifications of cell wall mechanics under stress conditions and the role of BSs in their regulation.

## 4. Materials and Methods

### 4.1. Plant Materials and Growth Conditions

*Arabidopsis thaliana* (L. Heynh.) wild-type Columbia-0 plants were grown on ES medium [18] with or without the addition of 100 nM 24-epibrassinolide (Sigma-Aldrich, St. Louis, MO, USA) diluted from a stock in absolute ethanol. Surface-sterilized seeds were sown aseptically on the medium disposed to 120 mm × 120 mm × 17 mm square Petri plates (Greiner Bio-One, Mosonmagyaróvár, Hungary) and stratified for 3 days at 4°. Their synchronous germination was induced by exposure to fluorescent white light (150 μmol m^−2^ s^−1^) for 4 h at 21 °C. The moment of transfer to light was taken as zero age for experimental plants. After the induction period, the Petri dishes were wrapped in four layers of aluminium foil and placed vertically in an environmentally controlled growth cabinet (cooled incubator BRC120, Bioconcept-Firlabo, Beun De Ronde, Drogenbos, Belgium, or ShSV-132 P, Termocon, St. Petersburg, Russia), and the plants were grown in darkness for 4 days at 21 °C.

### 4.2. Extensometry

*A. thaliana* seedlings were placed individually into 1.5 mL Eppendorf test tubes, frozen by immersing the closed tubes into liquid nitrogen, stored at −20 °C and used for biomechanical analyses within 2 weeks after freezing. In vitro extension of frozen/thawed hypocotyls was measured with a custom-built constant-load extensometer [10]. A 5 mm-long subapical segment (located 1.5 mm below the apical hook) of a four-day-old hypocotyl, including its growing zone [41], was secured between clamps of the extensometer and preincubated in a buffer (20 mM sodium acetate, pH 5.0, or 20 mM MES-KOH, pH 6.0) in a relaxed state for 2 min. Then, its time-dependent extension (creep) was measured in the same buffer under 400–700 mg loads for 15 min. The pH 5 buffer was used for creep measurements because it activates expansins that mediate the acid growth response [17]. Expansins are not active at pH 6, but this pH value is optimal for different cell-wall-loosening proteins including XTHs [39]. These pH values are within the physiological pH range for plant cell walls [42]. The relative creep rate was calculated as described in [7]. Before some creep tests hypocotyl cell walls were heat-inactivated at 90 °C for 3 min, as described in [19]. Creep of heat-inactivated cell walls is determined by physical properties of their polymers without ongoing modifications of their cross-linking by cell-wall-loosening/tightening proteins.

These experiments were conducted during a research stay at the university of Antwerp from 20 March 2014 to 19 May 2014.

### 4.3. Calculation of Cell Wall Cross-Section Area and Tensile Stress Under a Constant Load

The creep rate of hypocotyls is proportional to the stress (a force divided by the cross-section area across which it acts) in their cell walls resulting from the action of a constant load. To calculate this stress, the cross-section area of the hypocotyl cell walls was determined by measuring their dry weight per unit length [43], assuming that the wall density (*ρ*) is 1.5 g cm^−3^ [44]. By definition, *ρ* = *m*/*V*, where *m* is mass and *V* is volume. For a segment of a cylindrical organ like a hypocotyl *V* = *l* × *A*, where *l* is the length of the segment (5 mm) and *A* is its cross-section area. From the above equations, *A* = *m*/*(ρ* × *l*) and can easily be calculated using the known length of the hypocotyl segments (5 mm), their measured mass and assuming that *ρ* = 1.5 g cm^−3^.

Cell wall mass for calculating its cross-section area was measured using 5 mm-long segments (corresponding to those used in the creep test) excised from fresh *A. thaliana* hypocotyls with a custom-made double-bladed cutter. Eighty hypocotyl segments were transferred to a container for critical point drying (Microporous Specimen Capsules and Caps; 120–200 µm pores, Electron Microscopy Sciences (EMS); AURION, Wageningen, The Netherlands). Each container with hypocotyl segments was transferred to an individual borosilicate glass Petri plate, where the segments were extracted and dehydrated by four sequential 1 h washes in chloroform:methanol (1:1, *v*/*v*) followed by a 1 h wash in diethyl ether with subsequent air drying in a fume hood [19,33]. The weight of the dry wall material prepared from 80 5 mm-long segments of Arabidopsis hypocotyls was determined using a balance (SARTORIUS 2405, Göttingen, Germany) with a resolution of 1 μg. Then, the wall cross-sectional area was calculated using the above equation.

The wall stress (MPa) during the uniaxial extension of *A. thaliana* hypocotyls in vitro under a constant load was calculated as the ratio *F*/*A* (*F*: tensile force (N), *A*: hypocotyl cell wall cross-section area (m^2^)). The 400, 500, 600 and 700 mg loads used in the creep test generated tensile forces of 0.00392 N, 0.00490 N, 0.00588 N and 0.00687 N, respectively.

### 4.4. Calculation of Creep Rate × Stress^−1^, in Vitro Cell Wall Extensibility (ϕ) and in Vitro Cell Wall Yield Threshold (y) Values

Additional biomechanical characteristics creep rate × stress^−1^, in vitro cell wall extensibility (ϕ) and in vitro cell wall yield threshold (*y*) were calculated and statistically analyzed using scripts published in [19] that perform all these manipulations automatically after correct source data formatting. The script for creep rate × stress^−1^ (Script S1.R) and the one for ϕ and *y* (Script S2.R), along with instructions on their use, can be found as free downloads (Supplementary Material) at https://www.publish.csiro.au/FP/FP15190 (accessed on 1 January 2025).

As a numerator and a denominator in the ratio creep rate × stress^−1^ are independent variables, a bootstrap procedure was used to calculate this characteristic. Ten thousand bootstrap samples of size 4 were obtained by sampling with replacement from the available measurements of creep rate and wall stress. Comparison of the ratio estimates between groups were then performed using a Student’s *t*-test and, when needed, corrected for multiple comparisons by maintaining the false discovery rate at 5% [45].

The dependence of creep rate on wall stress was estimated by fitting Model II linear regression models with errors in both variables using the Maximum Likelihood Functional Relationship implementation from [46], a variant of Deming regression, assuming the residual standard error is proportional to the standard error of the sample. The code of the method is available at https://stat.ethz.ch/pipermail/r-help/2010-February/227865.html (accessed on 1 January 2025). In vitro cell wall extensibility was estimated as the slope of the regression line with stress as the independent and with creep rate as the dependent variable. The in vitro yield threshold was estimated by regressing stress as a dependent variable on creep rate as the independent variable and taking the intercept of this line. This is a valid approach because the Maximum Likelihood Functional Relationship method is symmetric and gives the same result, regardless of whether *x* is regressed on *y* or *y* on *x*, as pointed out in [46]. Non-parametric standard errors for the slope and the intercept were derived using leave-one-out jackknife.

Regression significance was determined by testing the significance of the slope coefficient, using the slope and standard error estimates to calculate a *t*-ratio and testing on a *t*-distribution with *N*-*p*-1 degrees of freedom where *N* is the number of loads and *p* is the number of predictors in the regression (1 for linear regression).

Differences between slopes and intercepts of regression models were tested by computing a test statistic (*t*-ratio) for the difference between the parameters of two models using the following formula:

*t* = (*coef*_1_ − *coef*_2_)/√0.5(σ_1_^2^ + σ_2_^2^),

where *coef*_1_ and *coef*_2_ are the respective standard errors of the estimates and testing on a two-tailed *t*-distribution with *df*_1_ + *df*_2_ degrees of freedom, where *df*_1_ and *df*_2_ are the degrees of freedom of the two regression models (two in all cases).

### 4.5. Transmission Electron Microscopy

Hypocotyls of 4-day-old *A. thaliana* seedlings were immersed in the fixative mixture containing 2.5% glutaraldehyde, 2% paraformaldehyde in 0.1 M cacodylate buffer, pH 7.2, overnight at 4 °C, postfixed with 1% OsO_4_ for 30 min on ice and then dehydrated in graded ethanol series and propylene oxide and embedded in epon EmBed812 resin according to recommendations of the manufacturer (Electron Microscopy Scienses, Hatfield, PA, USA). Transverse and longitudinal ultrathin (about 70 nm) sections were taken at 3 mm under the hypocotyl hook using ultratome EM UC 7 (Leica Microsystems, Wien, Austria) and a diamond knife (Diatome, Nidau, Switzerland) and mounted on formvar-coated copper grids. Sections were double stained with 2% uranyl acetate and Reynolds’ lead citrate [47]. The sections were observed, and images were taken using TEM Libra120 (Zeiss, Oberkochen, Germany). Three to five hypocotyls were sampled per cutting plane.

## 5. Conclusions

In summary, *Arabidopsis thaliana* hypocotyl growth inhibition by exogenous epibrassinolide (EBL) was accompanied by intriguing changes in cell wall biomechanics. EBL increased the wall viscoelasticity, while decreasing its extensibility. The former change was associated with cellulose microfibril disorganization in the presence of EBL, while the latter resulted from decreased activities of cell-wall-loosening proteins, presumably expansins. These adaptive changes in the wall biomechanics could enable plants to grow slowly under reduced turgor, helping them survive under stress conditions.

## Figures and Tables

**Table 1:** Tensile stresses generated in cell walls of hypocotyls from 4-day-old etiolated Col-0 *Arabidopsis thaliana* seedlings grown with 100 nM EBL or without it (control) in vitro under constant loads.

Characteristic	Control	EBL ^1^
Dry weight of one 5 mm-long segment (μg)	1.52 ± 0.11	2.30 ± 0.13
Calculated cell wall cross-section area (μm^2^)	202 ± 15	307 ± 17
Tensile stress from 400 mg load (MPa)	19.5 ± 1.5	12.8 ± 0.7
Tensile stress from 500 mg load (MPa)	24.4 ± 1.9	16.0 ± 0.9
Tensile stress from 600 mg load (MPa)	29.2 ± 2.3	19.2 ± 1.1
Tensile stress from 700 mg load (MPa)	34.1 ± 2.6	22.4 ± 1.3

^1^ All characteristics listed above were significantly different between EBL-grown plants and the untreated control (data are means ± SD; *n* = 4; *p* < 0.0001; Student’s *t*-test).

**Table 2:** In vitro cell wall extensibility (ϕ) and in vitro cell wall yield threshold (*y*) of hypocotyl cell walls from 4-day-old etiolated Col-0 *Arabidopsis thaliana* seedlings grown with 100 nM EBL or without it (control). The values of ϕ (±SE) correspond to slopes of the fitted straight lines in Figure 3. The values of *y* (±SE) correspond to intercepts of the fitted straight lines with the *x* axis in Figure 3. Different letters ‘a’ and ‘b’ mark significant difference in ϕ for the walls of control plants extended at pH 6 and pH 5 (*p* < 0.0001; Student’s *t*-test); different letters ‘c’ and ‘d’ denote significant difference in ϕ for the walls of control and EBL-grown plants extended at pH 5 (*p* < 0.0001; Student’s *t*-test); different letters ‘e’ and ‘f’ mark significant difference in *y* for the walls of control and EBL-grown plants extended at pH 5 (*p* < 0.0001; Student’s *t*-test); ND indicates Not Determined in cases where Deming regression was not significant.

	Control		EBL	
	ϕ (% h^−1^ MPa^−1^)	*y* (MPa)	ϕ (% h^−1^ MPa^−1^)	*y* (MPa)
pH 6	0.24 ± 0.03 ^a^	14.79 ± 1.26	ND	ND
pH 5	0.80 ± 0.02 ^b,c^	15.23 ± 0.22 ^e^	0.31 ± 0.02 ^d^	5.42 ± 0.47 ^f^
pH 5 heat-inactivated	ND	ND	ND	ND

## Data Availability

The data presented in this study are available on request from the corresponding author. The data are not publicly available due to privacy restrictions.

## Supplementary Materials

The following supporting information can be downloaded at: https://www.publish.csiro.au/FP/FP15190 (accessed on 1 January 2025).
